# Antecedents of Tourists’ Environmentally Responsible Behavior: The Perspective of Awe

**DOI:** 10.3389/fpsyg.2022.619815

**Published:** 2022-03-22

**Authors:** Juan Jiang, Bo Wendy Gao, Xinwei Su

**Affiliations:** ^1^Faculty of International Tourism and Management, City University of Macau, Macau, China; ^2^School of Tourism, Liming Vocational University, Quanzhou, China

**Keywords:** natural environment, awe, tourist satisfaction, SOR framework, tourists’ environmentally responsible behavior

## Abstract

The promotion of tourists’ environmentally responsible behavior (TERB) plays a central role in destination management for sustainability. Based on the stimulus–organism–response framework, this study proposes an integrated model for behavior management by examining the relationship between stimuli (natural environment and availability of infrastructure) and response factors (satisfaction and TERB) through the organism (the emotion of awe). Survey data from 458 tourists visiting Mount Heng in Hunan Province, Southern China, were used to empirically evaluate the proposed framework. The findings demonstrate that the perception of a destination’s natural environment positively impacts tourists’ sense of awe and satisfaction; the perception of availability of infrastructure positively and significantly influences awe, satisfaction, and TERB; and awe positively impacts satisfaction and TERB. Moreover, the emotion of awe plays a significant mediating role in this proposed model. The theoretical significance of this study and the implications for tourism destinations are discussed.

## Introduction

In 2018, the number of international tourists reached 1.4 billion, and it is estimated that this number will reach 1.8 billion by 2030 (United Nations World Tourism Organization [UNWTO], 2011). An aspect of this large-scale tourism, the reckless and inconsiderate behavior of tourists, contributes significantly to the many environmental problems experienced by tourism destinations, such as environmental pollution, damage to plant and animal habitats, and degradation of ecological resources and the environment (Lee et al., 2013; Zhang et al., 2019). For example, more than 200 million tourists visit the Mediterranean each summer, leading to a 40% increase in plastic waste in the sea (United Nations Environment Program [UNEP], 2019). Globally, it is estimated that 4.8 million tons of trash are produced by tourists each year. This means that destinations worldwide, from Stonehenge to Machu Picchu, from Mount Everest to Bali, are all struggling to deal with an increasing amount of trash left behind by tourists (Somani, 2019). Therefore, the sustainable development of tourist destinations has been facing very significant challenges.

Some destinations, such as the island of Boracay in the Philippines and Maya Bay in Thailand (United Nations Environment Program [UNEP], 2019), have been forced to close temporarily to recover from the pollution and other damage caused by tourists. However, for most destinations, especially those in developing countries, the tourism industry makes significant contributions to the GDP of the host countries and to employment opportunities. Therefore, halting tourism is not a straightforward matter. Moreover, as recognized by numerous researchers, to cope with the environmental challenges engendered by the fast-growing tourism industry, promoting tourists’ environmentally responsible behavior (TERB) would be the most workable and effective way to reduce the negative environmental impacts of tourism. This strategy would also promote the protection of destination resources, ultimately resulting in the sustainable development of those destinations (Wang X. et al., 2020). Thus, the important practical significance of TERB research to the long-term and sustainable development of tourist destinations and the factors that inspire TERB have resulted in widespread concern in recent years and are becoming an important research frontier (Cheng and Wu, 2015).

Given the importance of understanding the mechanism of the formation of TERB, multiple theories have been developed and applied in existing studies. These theories include planned behavior theory (Han et al., 2010), normative motivation theory (Gao et al., 2017), value–belief–norm theory (Van Riper and Kyle, 2014), social capital theory (Li and Wu, 2020), goal-directed behavior (Han and Yoon, 2015), and an integrated analysis framework of the theories mentioned above (Lindenberg and Steg, 2007; Han and Hyun, 2017). The variables covered by these theoretical models, such as behavior attitude, perceived behavior control, ascription of responsibility, awareness of consequences, personal norms, and interpersonal trust, and the variables included in the extended model and framework, such as environmental attitude and tourism experience (Lee and Jan, 2015), environmental concern (Li and Wu, 2020), conservation commitment (Lee, 2011), and place attachment (Qu et al., 2017), focus more on personal intrinsic psychological factors than on contextual factors.

However, TERB as a sustainable behavior of an individual under unusual circumstances will not only be affected by the intrinsic attitude factors of tourists but also by external situational variables (Guagnano et al., 1995). Thus, compared with other theories and variable factors, the stimulus–organism–response (SOR) theory proposed in this study to understand the formation mechanism of TERB may be more effective in explaining tourist behavior. In addition to emphasizing the importance to TERB of situational factors, SOR also takes into account the driving effect on TERB of the positive emotions generated by the individual. However, when the original theoretical model was used to explain the formation of TERB, it was limited to the original variables in the model, making it difficult to select more effective variables according to the research situation. Based on the original theoretical chain, SOR theory can select research variables and explore the causal relationship between them according to the specific research situation, thereby explaining the tourist’s response behavior more effectively.

Therefore, this study proposed SOR theory as the theoretical basis to explore the antecedents of TERB in a mountain-based natural destination. Other forms of tourism, such as eco-tourism, emphasize the tourism motivation of responsibility for the environment and the economy (Chiu et al., 2014). Urban tourism involves broader environmentally responsible behaviors that stress the importance of habit (Miller et al., 2015). Cultural tourism focuses on the authenticity of its tangible and intangible qualities that arouse awe (Wang E. et al., 2020). Those who participate in mountain-based natural tourism are attracted by the impressive mountain scenery and are motivated by the intrinsic emotion derived from the contextual factors to engage voluntarily in TERB. Among the emotions that tourists experience, the emotion of awe is one of the most anticipated experiences for tourists in mountainous landscapes (Powell et al., 2012). This emotion can stimulate a close connection between the tourists and their environment (Pearce et al., 2017), prompting them to show positive local protective behaviors (Coghlan et al., 2012). Therefore, based on the SOR theory, it is meaningful and valuable to introduce the concept of awe and to explain the internal mechanism of the formation of tourists’ TERB.

Arising from the preceding discussion, we propose an integrated model based on SOR theory, which considers the contextual factors (natural environment and availability of infrastructure) as the stimuli, awe as the organism, and tourists’ satisfaction and environmentally responsible behavior (ERB) as the responses. We anticipate that the contextual factors may not only affect satisfaction and TERB directly (Cheng et al., 2013; Chubchuwong et al., 2015) but may even facilitate tourists’ behavior response by stimulating their emotion (Wang et al., 2019), manifested as the perception of awe. In addition, the application of the SOR framework in the existing TERB literature has been limited (Su and Swanson, 2017; Su L. et al., 2020), and few researchers have considered the emotion of awe and contextual factors as antecedents of TERB (Coghlan et al., 2012; Wang and Lyu, 2019; Wang et al., 2019). Therefore, the conceptual framework we propose is necessary and valuable to understand the formation of the response of tourists to a destination. Thus, by exploring the SOR-based formation mechanism of satisfaction and TERB, this study will not only extend the application of the existing theory of awe and the SOR framework but will also provide suggestions for the sustainable management and development of destinations.

## Theoretical Foundation and Hypothesis Formulation

### Stimulus–Organism–Response Framework

The SOR framework, first proposed by Mehrabian and Russell (1974), demonstrates that an internal state (O) is generated when an individual is exposed to external stimuli (S), subsequently dictating the responses of the individual (R). Specifically, stimuli, including object stimuli and social-psychological stimuli, will help an individual to elicit his or her internal cognitive and emotional states, thereby triggering a response of either approach or avoidance (Lee et al., 2011). The validity of the SOR model, as a parsimonious and robust framework in predicting individuals’ responses, has been verified by studies in multiple settings, such as technology products (Lee et al., 2011), tourism destinations (Su and Swanson, 2017), hotel human resource management (Su and Swanson, 2019), and restaurant consumption experiences (Jang and Namkung, 2009).

Individuals receive not only object stimuli but also social-psychological stimuli as environmental stimuli (Slama and Tashchian, 1987). Thus, in the context of a tourist destination, stimuli include tourists’ perceptions of the physical environment (e.g., natural environment) surrounding them and how the destination is managed (e.g., the availability of infrastructure) (Su L. et al., 2020). When tourists are exposed to the object stimuli (natural environment) and social stimuli (availability of infrastructure) of the destination, an intrinsic positive emotion (e.g., awe) (Pearce et al., 2017) is evoked, and subsequently, tourists’ responses change in response to that emotion. It is noteworthy that no complete consensus has been reached as to whether satisfaction is an organism variable or a response variable (Su L. et al., 2020). Although the marketing and tourism fields generally consider that satisfaction is the psychological state generated by the stimulation of a consumer experience, most empirical research models based on the SOR paradigm tend to consider satisfaction as a response component (Eroglu et al., 2003; Mummalaneni, 2005) because of its post-purchase attitude characteristic (Swan and Combs, 1976). For instance, in the empirical model of Eroglu et al. (2003), satisfaction, as with approach/avoidance behaviors, was considered the outcome variable of the consumption process. It was also specifically regarded as the response component of the SOR framework. Additionally, in the two studies by Mummalaneni (2005) and Su L. et al. (2020), satisfaction was also classified as a component of “response” rather than of “organism” in their proposed viable models of the SOR framework. Hence, based on these empirical studies, the present study proposes satisfaction as a response variable and an outcome variable.

**FIGURE 1:**
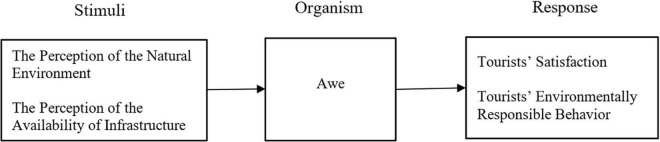
The SOR model proposed in this study.

In our model, satisfaction and TERB, as components of response (response), are triggered by tourists’ emotional state of awe (organism) which, in turn, is generated from perceptions of the natural environment and the availability of infrastructure (Stimuli) (Figure 1).

### Stimuli: Natural Environment and the Availability of Infrastructure

The term “natural environment,” sometimes used interchangeably with the term “nature,” refers to “an environment with little or no apparent evidence of human presence or intervention” (Hartig et al., 2014). In the tourism field, the natural environment refers to the environment of the destination formed by natural resources, such as natural creatures (e.g., animals and plants), natural scenery (e.g., landforms, mountains, coasts, beaches, and oceans), and other natural attractions (e.g., weather) (Ross and Iso-Ahola, 1991; Lu et al., 2017). In the present study, the perception of the natural environment is considered to be the cognitive affiliation with nature (Basu et al., 2020) and a subjective evaluation of the natural features of a destination (Hartig et al., 2014).

First, earlier research literature discussed the causal relationship between contact with the natural environment and the experience of awe. For example, Ballew and Omoto (2018) found that compared to built environments, natural environments significantly enhanced feelings of awe and other positive emotions. More importantly, compared with other positive emotions, such as amusement and joy, the connection between the experience of awe and the perception of the natural environment was significantly stronger (Anderson et al., 2018). Joye and Bolderdijk (2015) showed that, when participants are shown an awe-inspiring natural slideshow, they usually feel small and humble, feelings that are the central appraisals of the experience of awe. In exposure to and connectedness with nature, the feeling of awe is an intense emotion, among other transcendent emotions triggered by nature (Bethelmy and Corraliza, 2019). It is noteworthy that in tourism, individuals’ perceptions of the extraordinary natural environment are likely to elicit emotional reactions, such as awe (Powell et al., 2012). Therefore, in this study, we anticipated that tourists’ perception of the destination’s spectacular mountain scenes would have a positive impact on their experience of awe.

Second, previous studies discussed the direct relationship between tourists’ perception of a destination’s natural environment and their satisfaction. For example, the structural equation modeling (SEM) analyses of Chi and Qu (2008) and Lu et al. (2015) found that tourists’ evaluation of a destination’s natural attractions (e.g., scenic mountains and valleys, breathtaking scenery) significantly impacts their overall travel satisfaction. The finding of Chi and Qu (2008) further verified that tourists’ satisfaction with natural attractions leads to their satisfaction with the tourism experience. The more that tourists appreciate the natural phenomena and scenery of a destination, the more satisfied they will be. The empirical research results of Buckley et al. (2014) further confirmed this conclusion in the context of river-based adventure tourism. Satisfaction with the nature of a destination is one of the important indicators of tourist satisfaction with the overall travel experience. In addition, the results of a quantitative study by Lu et al. (2017) indicated that the perception of an extraordinary natural landscape of a mountain destination was found to help improve tourists’ levels of satisfaction. Thus, it is reasonable to infer that the tourists’ perception and evaluation of the natural attractions of a destination will have a positive effect on their satisfaction.

Third, a close relationship between individuals and the natural environment was found to be conducive to general ERB in daily life in theory and correlation research (Vaske and Kobrin, 2001). For example, compared with people who spend more time in artificial environments (such as zoos), people who are often exposed to the natural environment are more likely to have attitudes and behaviors that reinforce environmental protection (Stewart and Craig, 2001). The results of three experimental studies by Zelenski et al. (2015) suggested that participants who were exposed to nature videos expressed stronger willingness to engage in environmentally sustainable behaviors. Moreover, the natural scenery of a tourist site, as part of the environmental background, usually encourages tourists to engage in valuable environmentally protective behaviors (Wang et al., 2019). Thus, given the significance of the individual’s exposure to nature to their ERB, it would, therefore, be reasonable to infer that the perceived evaluation of the natural environment influences TERB. Accordingly, we propose the following hypotheses:

H1: The perception of the natural environment is positively related to the experience of awe.
H2: The perception of the natural environment is positively related to tourists’ satisfaction.
H3: The perception of the natural environment is positively related to TERB.

The availability of infrastructure, not only refers to the basic facilities that tourists rely on to enjoy the tourism experience, in that, affect tourists’ behavior of repeat visit and recommended to others (Sannassee and Seetanah, 2014); more importantly, it refers to the availability of pro-environment information, products, and facilities (Chubchuwong et al., 2015; Miller et al., 2015) that influence tourists’ environmental attitudes and behaviors. Our search of the literature^1^ showed that no empirical study had investigated the direct relationship between the perception of availability of infrastructure and awe and satisfaction. However, there have been findings on the relationship between ecological and environmental management variables (including destination and corporate contexts) and emotional experience. For example, Su L. et al. (2017) and Su and Swanson (2019), respectively, surveyed employees and guests of hotels in China and confirmed that the employees’ or guests’ perceptions of corporate social responsibility (including environmental corporate social responsibility) directly affect their emotional responses to the company in the form of trust, recognition, and positive emotions. Han et al. (2019) revealed that emotional factors, respect, and preference for brands are significantly influenced by customers’ perceptions of the environmental corporate social responsibility of airlines. In addition, according to studies by Su and Swanson (2017) and Su L. et al. (2020), tourists experience positive emotions because their reactions will be triggered by the implementation of ecological and environmental practices in a destination or by the environmentally responsible operation of the destination.

In this study, the destination’s environmental efforts (such as public transportation systems) facilitate access for most tourists (such as those who cannot reach the summit on foot) and remove barriers to experiencing a sense of awe. Other practices (such as environmentally responsible tourism programs and information) can directly evoke a sense of awe in tourists because they focus on the harmony between man and nature and the future sustainability of natural resources, which, in turn, can make tourists feel small (Piff et al., 2015). Therefore, awe is proposed here as a form of positive emotion and is expected to be influenced by tourists’ evaluations of the destination’s eco-friendly facilities like other positive emotional responses.

Similarly, a good mall or shopping environment will positively affect customer satisfaction during the consumption process (Walsh et al., 2011). In a tourism study, Kwek et al. (2014) found that the overall level of satisfaction of groups of overseas tourists visiting China was affected by transport, tourist sites, and accommodation. Therefore, as an important aspect of the environment in which the tourist experience is consumed, the availability of infrastructure may also affect tourist satisfaction to the same extent as any other environmental factor. If a tourist considers that the destination provides sufficient environmental protection facilities, services, and programs, and that the destination has a good eco-friendly reputation, he or she will be more satisfied with his or her travel experience (Su L. et al., 2020).

Similar to the relationship between the natural environment and TERB, the availability of infrastructure as a stimulus may also directly affect TERB as a response. Previous studies found that if a destination does not have sufficient environmentally friendly services and facilities available, tourists may be hindered from implementing specific ERBs (Miller et al., 2015). Moghimehfar and Halpenny (2016) also confirmed that facility constraints at campsites directly and negatively influence the ERB intentions of campers. Also, in studies of international visitors to Thailand, Chubchuwong et al. (2015) found that the lack of availability of “green” infrastructure, products, and information had a negative effect on tourists’ attitudes toward environmental protection and on their behaviors. Therefore, it is only when basic environmental protection facilities, such as recycling bins and public transport systems, are provided that tourists will adopt correct environmental behaviors (Miller et al., 2015). Combined with the findings of studies in the literature, we hypothesize that:

H4: The perception of the availability of infrastructure is positively related to the experience of awe.
H5: The perception of the availability of infrastructure is positively related to tourists’ satisfaction.
H6: The perception of the availability of infrastructure is positively related to TERB.

### Organism: Awe

Awe is an emotional response to the comprehensive perception of the tourism experience generated in tourists as a response to a variety of destination factors, like the natural environment, sacred sites, majestic architecture, celebrity-related places, and other attractions of tourist destinations (Keltner and Haidt, 2003; Coghlan et al., 2012). As an intense positive emotion that individuals often experience when visiting nature-based destinations (Pearce et al., 2017), awe is an emotion that tourist products and tourist destinations strive to elicit (Coghlan et al., 2012). However, studies of tourism-generated awe are scarce (Pearce et al., 2017). The limited existing studies are, to a large extent, exploratory studies of awe and how it is elicited (Farber and Hall, 2007; Davis and Gatersleben, 2013). Few studies specifically examine the relationship between awe and tourists’ levels of satisfaction and their resulting behaviors (Lu et al., 2017; Wang and Lyu, 2019).

According to H1 and H4, the intrinsic state of awe of tourists is evoked by stimuli of the natural environment and the availability of infrastructure. This state may exert an impact on tourists’ levels of satisfaction and their resulting behaviors. First, the connection between awe and tourist satisfaction is based on the link between positive emotions and satisfaction. Su and Hsu (2013) conducted an empirical study on Chinese heritage tourists and found that positive emotions have a direct positive impact on tourist satisfaction. Second, the participants in the experiment who experienced awe also experienced greater life satisfaction in general (Rudd et al., 2012). Third, in the study by Powell et al. (2012) of tourists’ perception, evaluation, and emotion of their Antarctic tourism experience, a positive correlation was confirmed between awe and satisfaction. In an investigation of 296 tourists who visited Mount Emei in Sichuan Province, Lu et al. (2017) also found that the effect of the experience of awe on tourists’ levels of satisfaction was significant.

In addition, as a positive emotion with a transformative function, awe can strengthen the close connection between individuals and their environment, thereby generating prosocial motivation (Piff et al., 2015; Pearce et al., 2017). Most environmental protection behaviors are also prosocial. Therefore, there is a close connection between feelings of awe toward nature and environmentally protective behaviors (Zelenski and Desrochers, 2021). Taking college students as the experimental participants, Yang et al. (2018) proved that awe induced not only by natural scenery but also by powerful people could promote environmentally beneficial behaviors among the research subjects. Furthermore, based on three studies in the context of eastern and western cultures (including an on-site survey and two experimental studies), Wang and Lyu (2019) verified that the sense of awe induced by the travel experience could encourage tourists to implement ERBs by reducing their self-concern. Based on the above discussion, we hypothesize that:

H7: The experience of awe is positively related to tourists’ satisfaction.
H8: The experience of awe is positively related to TERB.

### Response: Tourist Satisfaction and Tourists’ Environmentally Responsible Behavior

#### Tourist Satisfaction

Tourist satisfaction, proposed as one response component in this study, has been widely studied in tourism for its importance in repurchasing, recommending, or revisiting (Choo and Petrick, 2014; Lee et al., 2014). In the tourism context, the first proposed, and also the prevailing definition of satisfaction, is “the result of the interaction between a tourist’s experience at the destination area and the expectations he (*sic*) had about that destination” (Pizam et al., 1978). This definition is inclined toward overall satisfaction, the more important type of the two common formulations of satisfaction due to its more profound and valuable role in predicting tourists’ behavioral intentions and the future performance of tourism enterprises (Eid and El-Gohary, 2015). Therefore, satisfaction, as referenced in this study, refers to the overall formulation, as in the case in almost all satisfaction studies (Anderson et al., 1994).

However, due to the rapid development of mass tourism in recent decades, most popular tourist destinations have become overcrowded, especially in developing countries and regions that rely heavily on the tourism industry for revenue. This consideration means that it is vitally important to assess visitors’ overall travel experience in these destinations. This is because the over-development of tourist destinations will, to a certain extent, prompt tourists to reevaluate their experience. Therefore, given the influence of the perception of the destination’s core and augmented attributes on visitor’s attitudes and behaviors (Cheng et al., 2013; Buckley et al., 2014), we propose to explore tourists’ satisfaction associated with the stimuli factors of destinations. Based on the previous discussion, the natural environment and the availability of infrastructure are explored in this study. The intention is to confirm the extent to which these factors contribute to meeting tourists’ travel expectations in the context of the rapid development of tourism.

#### Tourists’ Environmentally Responsible Behavior

Tourists’ environmentally responsible behavior refers to the behaviors practiced by tourists “who strive to reduce environmental impacts, contribute to environmental preservation and/or conservation efforts, and not disturb the ecosystem and biosphere of a destination” (Lee et al., 2013) in the process of recreation/tourist activities. The term is usually used interchangeably with other terms such as pro-environmental behavior, environmentally concerned behavior, environmentally significant behavior, sustainable behavior, and eco-friendly behavior (Kiatkawsin and Han, 2017). Previous studies indicated that fostering TERB plays a central role in managing the sustainability of destinations (Wang X. et al., 2020) and appears to be the best practice for maintaining the latter (Lee et al., 2013).

Considering the importance of TERB for sustainability, existing studies have investigated the TERB in various fields of tourism, such as destination TERB (Cheng et al., 2013), hotel TERB (Han and Yoon, 2015), event TERB (Mair and Laing, 2013), cruise TERB (Han et al., 2016), and museum TERB (Han and Hyun, 2017). The antecedents covered aspects ranging from demographic to internal psychological factors, from contextual to habitual factors, and they also incorporated various mature theoretical and modified models to provide a comprehensive understanding of TERB (Van Riper and Kyle, 2014; Kiatkawsin and Han, 2017). In brief, given that TERB is not only important for the sustainable development of tourism (Kafyri et al., 2012) but also has a positive impact on the ERB of daily life (Ramkissoon et al., 2012), the investigation of the potential determinants that would prompt tourists to implement ERB will become increasingly important.

Therefore, this study focuses on the emotion of awe and other external stimulus factors as the antecedents of TERB that have been ignored to a certain extent. This approach is not only an innovative application of the SOR framework based on the experience and perception of awe but also an effective supplement to the existing research on the driving factors of TERB.

### Mediating Effect

Previous studies have found that the relationship between the natural environment and satisfaction is linked by the experience of awe. Specifically, awe has been found to partially mediate this relationship (Lu et al., 2017). However, to the best of our knowledge, there are no published studies that have investigated the role of awe in the relationship between the natural environment and TERB, the availability of infrastructure and satisfaction, or the availability of infrastructure and TERB. According to the SOR framework, there should be a connection between the external factors of a destination and satisfaction/TERB through the emotion of awe. For example, building on the SOR framework (Mehrabian and Russell, 1974), Su and Swanson (2017) and Su L. et al. (2020) proposed that tourists’ emotional responses during an experience are generated by the external stimuli of the destination itself and that tourists then exhibit different behaviors. Tourists experiencing positive emotions are more likely to adopt behaviors that follow social norms. Subsequently, these researchers found that tourists’ positive emotions (organism) were elicited by information about the eco-friendly reputation of a destination or its sense of social responsibility (stimuli), leading to tourist satisfaction and ERB (response).

Therefore, it is reasonable to consider that, just as with a destination’s eco-friendly reputation and sense of social responsibility, other external factors of the destination, in this study, specifically referring to the natural environment and the availability of infrastructure, may also affect tourist satisfaction and TERB through the intermediary effect of the perception of awe. Based on this discussion, the following hypotheses are proposed:

H9: The experience of awe mediates the effect of tourists’ perception of the natural environment (a) and the perception of the availability of infrastructure (b) on tourists’ satisfaction.
H10: The experience of awe mediates the effect of tourists’ perception of the natural environment (a) and the perception of the availability of infrastructure (b) on TERB.

**FIGURE 2:**
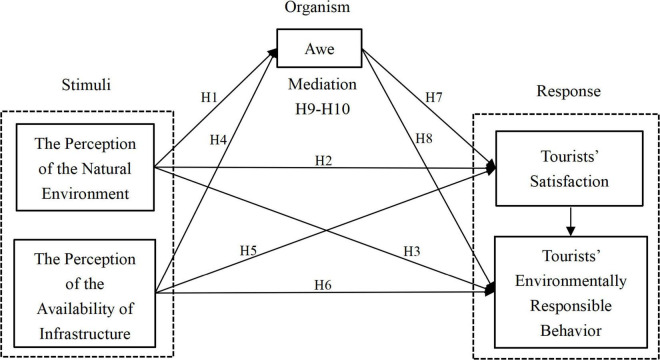
A conceptual framework of the SOR model.

Based on these hypotheses, Figure 2 provides a conceptual framework for the present study, which includes the hypotheses and the relationships between the five constructs. More importantly, in the field of TERB research, this study is the first to empirically consider awe as a connection between external stimuli and the responses of tourists.

## Materials and Methods

### Study Area

**FIGURE 3:**
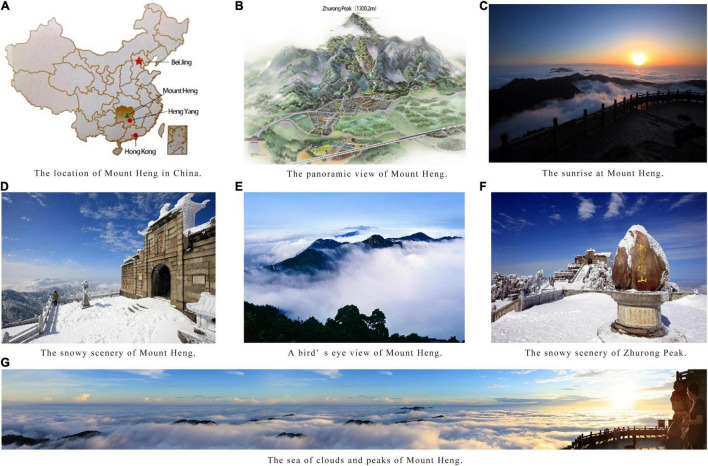
The location **(A)** and natural scenery **(B–G)** of Mount Heng. Source: Tourism administration of Nanyue District.

In the traditional culture of China, Mount Heng, regarded as the Southern Great Mountain, together with the Eastern Great Mountain, the Western Great Mountain, the Northern Great Mountain, and the Central Great Mountain, is known as one of the sacred mountains. Of the five sacred mountains, Mount Heng is located in south-central Hunan Province and is the only one in the south of China (Figure 3). It is known for its beautiful scenery and long history and was included in the Natural and Cultural Heritage List of China in 2006 for its excellent natural and religious cultural resources. It is also famous for its more than 200 Buddhist and Taoist temples, and for the god of Mount Heng, Zhurong, the god of fire. Zhurong Peak (1,300.2 m) is the main and the highest peak of the 72 peaks of Mount Heng, named in memory of Zhurong. Mount Heng refers to the scenic area with Zhurong Peak at its center. Every year, millions of tourists go to Mount Heng to watch the sunrise, see the sea of clouds, and enjoy the snowy scenery and the beautiful peaks (Figure 3). The emotion of awe is easily inspired in tourists by the magnificent and impressive natural scenery.

According to the annual report released by the Hengyang City Statistics Bureau for the period from 2010 to 2019, millions of tourists visit the mountain every year, with more than a hundred thousand visitors per day during the peak season. For example, on the “May Day” and “National Day” holidays in 2019, there were more than 160,000 tourists and 600,000 tourists, respectively, resulting in enormous challenges to the environmental protection practices of the scenic area and affecting the satisfaction experience of tourists. As a response to the pressure brought to bear by tourism development, the local authorities formulated the “Regulations on the Protection of Mount Heng,” strengthened the daily maintenance of environmental protection and cleanliness and provided environmentally friendly transport for tourists. Additionally, a natural protection public center was established to engage in special pro-environment activities, such as tree planting, preservation of ancient roads, and environmental cleaning. These environmental protections are effective. The improvements bring more tourists into close contact with the destination by reducing the number of private vehicles, allowing larger numbers of visitors to access and appreciate the magnificent natural wonders. In addition, the new environmental protection activities help preserve the scenic locale and ensure the quality of tourism experience.

Therefore, Mount Heng was chosen as the study area because it is a representative nature-based tourist destination. More importantly, it is a suitable site for our proposed model, which utilizes awe as the mediating variable that explains the effect of the perception of the natural environment and the availability of infrastructure on tourists’ satisfaction and TERB.

### Questionnaire Design and Instrument Development

The questionnaire consisted of two parts. The first part included 24 items designed to measure the five constructs of the model. All scales except for awe were based on a seven-point Likert scale, from “strongly disagree (1)” to “strongly agree (7).” A semantic difference scale was used to measure awe. The second part of the questionnaire related to demographic information.

The scales for evaluating each variable were developed from previous studies. The scales for natural environment (four items), awe (four items), and tourist satisfaction (three items) were based on the study by Lu et al. (2017) in which the study context was similar to the present study. The scale of availability of infrastructure (seven items) from the study by Chubchuwong et al. (2015) was also included. Finally, the six items used to measure TERB were adapted from the study by Su and Swanson (2017). Table 2 shows the questions included; some of which were modified slightly to adapt them to the context of the present study.

The questionnaire was in Chinese (Mandarin) because most of the tourists to Mount Heng are Chinese. Following the guidelines recommended by Brislin (1970), the Chinese questionnaire derived from the original English scales was back translated to the English version to ensure that the translated version accurately expressed the original semantics.

### Sampling and Data Collection

Tourists who had just completed a visit to Mount Heng were surveyed at the main exit of the scenic spot. The two places (the tourist service center and the leisure shopping street) where tourists who have finished their tourism experience can rest are next to the main exit. Both places provide a large number of seats for tourists to facilitate their short stay. Therefore, the tourists who chose to rest had sufficient time to complete the questionnaire, and they were not under pressure to leave.

On December 10, 2019, before the formal survey, a face-to-face pilot study of the questionnaire was conducted at Mount Heng to evaluate its content validity. Thirty tourists participated in this survey, and all of them completed the questionnaire within 5 min. According to the feedback, none of them had any questions about the content and design of the questionnaire. We conducted our data collection from January 11 to 20, 2020, over 6 working days and 2 weekend holidays. Self-administrated questionnaires were distributed randomly in the peak periods during weekdays (3:00 p.m. to 6:00 p.m.) and weekends (2:00 p.m. to 6:00 p.m.). The tourists were asked whether they were willing to complete a questionnaire. They filled in the questionnaire voluntarily under the premise of knowing the academic purpose of the questionnaire. The number of questionnaires distributed every day was limited to ensure a wide range of tourists, thereby minimizing the bias caused by convenience sampling. On weekdays, 45 questionnaires were distributed per day, and on weekends, 60 questionnaires were distributed per day. In all, 510 questionnaires were distributed, 497 were returned, and there were 458 valid questionnaires.

## Results

### Demographic Information

Among the 458 respondents, over half (55.9%) were female, and a large proportion (71.9%) of the participants were aged between 18 and 35. This may have been because of the nature of the destination, a mountain area tends to attract younger tourists. Nearly 90% of the respondents had junior college education or above. More than 80% of the respondents were individual tourists. Almost half of the respondents had visited the destination previously. Detailed demographic information is presented in Table 1.

**TABLE 1:** Background information about the respondents (*N* = 458).

Demographic	Frequency	Percentage (%)
**Gender**		
Male	202	44.1
Female	256	55.9
**Age group**		
18–25	167	36.5
26–35	162	35.4
36–45	98	21.4
46–55	24	5.2
≥56	7	1.5
**Education**		
Middle school	54	11.8
Junior college	132	28.8
Undergraduate	244	53.3
Postgraduate	28	6.1
**Travel type**		
Group	85	18.6
Individual	373	81.4
**Income level (RMB)**		
≤ 3000	82	17.9
3001–6000	136	29.7
6001–10000	171	37.3
≥ 10,001	69	15.1
**Frequency of visit**		
1	231	50.4
2	127	27.7
3–4	58	12.7
≥5	42	9.2

### Results of Statistical Analyses

The SmartPLS 3.2.8 software for variance-based SEM using the partial least squares (PLS) path modeling method was employed to explore the causal relationships between stimuli, organism, and response factors. The PLS method is more reliable and efficient when processing non-normal distribution data (Hair et al., 2011) and when working with a small sample size because of its powerful statistical analysis functions. The conceptual model was analyzed in two steps in line with the instructions of Hair et al. (2017).

#### Measurement Model Assessment

First, the reliability and validity of the measurement model were evaluated. Table 2 shows the factor loadings, Cronbach’s α values, the average variance extracted (AVE), and the composite reliability (CR) of the constructs. The items “information about public transport” and “environmentally friendly transportation” were deleted due to a low factor loading (<0.60). The factor loadings of all other items were greater than 0.7, which is satisfactory. CR (>0.7) and Cronbach’s α (>0.7) were used to evaluate reliability, indicating a gratifying internal consistency of the five constructs (Hair et al., 2017). AVE was used to assess convergent validity. All values of AVE exceeded 0.5, suggesting that the convergent validity was satisfactory.

**TABLE 2:** Construct reliability and convergent validity.

Item	Factor loading	Cronbach’s α	CR	AVE
**The Perception of the Natural Environment**		0.785	0.861	0.609
Mount Heng shows me how strong the nature is	0.786			
Mount Heng impresses me with its majestic and precipitous appeal	0.782			
I feel Mount Heng is magnificent	0.839			
Mount Heng gives me a fantastic display of many beautiful peaks	0.710			
**The Perception of the Availability of Infrastructure**		0.813	0.869	0.572
Information on how to act responsibly toward the environment	0.758			
Environmentally responsible tour programs	0.793			
Environmentally responsible food and drink containers	0.745			
Normal garbage bins	0.703			
Recycling garbage bins	0.779			
**Awe**		0.800	0.870	0.627
Boring–exciting	0.863			
Usual–unusual	0.820			
Expected–unexpected	0.711			
Arrogant–humbling	0.765			
**Tourists’ Satisfaction**		0.839	0.903	0.757
In general, this site was much better than I expected	0.855			
This visit was well worth my time and effort	0.874			
Overall, I was very satisfied with my holiday at Mount Heng	0.880			
**Tourists’ Environmentally Responsible Behavior**		0.848	0.887	0.568
When I see garbage and debris at the Mount Heng destination, I put it in the trash	0.746			
I comply with the rules so as to not harm the environment of Mount Heng	0.791			
I try to convince others to protect the natural environment at the Mount Heng destination	0.774			
If there are environmental improvement activities at the Mount Heng destination, I am willing to attend	0.737			
I try not to disrupt the fauna and/or flora when visiting the Mount Heng destination	0.756			
I report to the appropriate destination administration any environmental pollution or destruction at the Mount Heng destination	0.716			

As shown in Table 3, the square root of all AVE variables was greater than its correlations with other variables. This is one method of confirming discriminant validity (Gefen and Straub, 2005). The second method is based on the value of the heterotrait–monotrait ratio of correlations (HTMT). All values of HTMT were less than 0.85, indicating that the discriminant validity was satisfactory (Henseler et al., 2015). In addition, all values of the variance inflation factor (VIF) were between 1.266 and 2.123, indicating that multicollinearity was not an issue (Becker et al., 2015).

**TABLE 3:** Correlation matrix of all variables.

Variable	AI	AWE	NE	SA	TERB
AI	**0.756**				
AWE	0.432 (0.534)	**0.792**			
NE	0.459 (0.568)	0.543 (0.682)	**0.781**		
SA	0.401 (0.478)	0.445 (0.540)	0.554 (0.680)	**0.870**	
TERB	0.502 (0.591)	0.412 (0.489)	0.372 (0.450)	0.492 (0.573)	**0.754**

*NE, the perception of the natural environment; AI, the perception of the availability of infrastructure; AWE, the experience of awe; SA, tourists’ satisfaction; TERB, tourists’ environmentally responsible behavior. The bold diagonal elements are the square roots of each AVE value; variable correlations are shown off-diagonal. The HTMT ratios are shown in parentheses.*

#### Structural Equation Model

**FIGURE 4:**
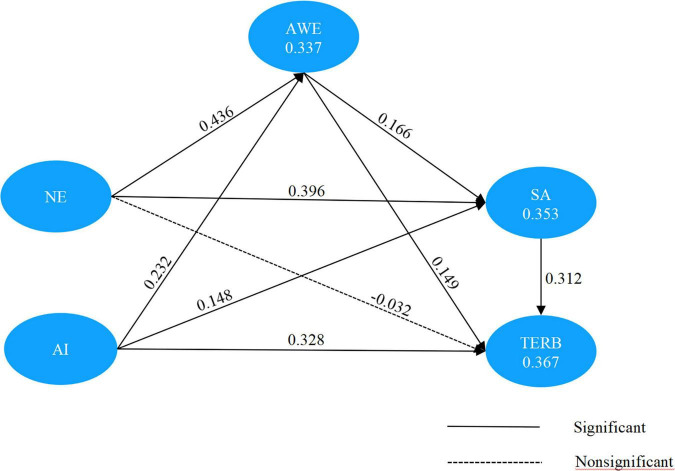
Completely standardized path coefficients among NE, AI, AWE, SA, and TERB. NE, the perception of the natural environment; AI, the perception of the availability of infrastructure; AWE, the experience of awe; SA, tourists’ satisfaction; TERB, tourists’ environmentally responsible behavior.

*R*^2^ and *Q*^2^ were used to measure the relationship of a latent variable’s explained variance to its total variance and the extent to which each prediction was successful, respectively (Urbach and Ahlemann, 2010). As shown in Figure 4, the *R*^2^ values of endogenous variables (awe: 0.337; satisfaction: 0.353; TERB: 0.367) were all greater than the definition of the critical value of *R*^2^ (low: 0.25; medium: 0.50; high: 0.75) (Hair et al., 2011), and the *Q*^2^ values were all greater than 0 (AWE: 0.197; satisfaction: 0.249; TERB: 0.190), indicating that the dependent variables are well explained, and the predictive power of the structural model is satisfactory (Urbach and Ahlemann, 2010).

In the second step of the assessment of the structural model, a bootstrapping resampling approach (3,000 samples) was used to evaluate the path coefficients between the model’s latent variables. The results of the model analysis (see Table 4 and Figure 4) show that natural environment perception significantly and directly influenced tourists’ awe (β = 0.436, *p*-value < 0.001) and satisfaction (β = 0.396, *p*-value < 0.001). Therefore, H1 and H2 are supported. This means that the perception of the natural environment of a destination is an important antecedent variable in the generation of tourists’ sense of awe and satisfaction. The perception of availability of infrastructure significantly influenced tourists’ awe (β = 0.232, *p*-value < 0.001), satisfaction (β = 0.148, *p*-value < 0.01), and TERB (β = 0.328, *p*-value < 0.001). Therefore, H4, H5, and H6 are supported. In light of these results, the more sufficient and available the tourists perceived the infrastructure, the stronger their awe and satisfaction, and their TERB increased. Awe positively and significantly affected satisfaction (β = 0.166, *p*-value = 0.001) and TERB (β = 0.149, *p*-value < 0.01). Therefore, H7 and H8 are supported. The results show that tourists who experienced awe are more likely to have a high level of satisfaction and TERB. However, H3, that TERB was directly influenced by the perception of the natural environment, was rejected (β = −0.032, *p*-value = 0.536), indicating that perception of the natural environment is not necessarily an antecedent variable of TERB.

**TABLE 4:** Results of the structural model.

Hypothesis	Path	Original sample	Standard error	*t*- value	*p*- value	Support
H1	NE → AWE	0.436	0.045	9.721	0.000	Yes
H2	NE → SA	0.396	0.055	7.180	0.000	Yes
H3	NE → TERB	−0.032	0.052	0.619	0.536	No
H4	AI → AWE	0.232	0.050	4.628	0.000	Yes
H5	AI → SA	0.148	0.049	2.989	0.003	Yes
H6	AI → TERB	0.328	0.048	6.861	0.000	Yes
H7	AWE → SA	0.166	0.050	3.330	0.001	Yes
H8	AWE → TERB	0.149	0.052	2.863	0.004	Yes

*NE, the perception of the natural environment; AI, the perception of the availability of infrastructure; AWE, the experience of awe; SA, tourists’ satisfaction; TERB, tourists’ environmentally responsible behavior.*

#### Mediation Effect

The bootstrapping method (3,000 samples) was also utilized to test the mediation effect of the sense of awe to explore further the relationship between the perception of the natural environment and TERB. The results of PLS analysis on the mediating effects indicate (see Tables 4, 5) that H9 and H10 are both supported. Given the non-significant direct relationship between the perception of the natural environment and TERB, awe fully mediates the effect of the perception of the natural environment on TERB (β = 0.065, *p*-value < 0.01). Therefore, although a direct effect does not exist, the effect of the perception of the natural environment on TERB is assumed from the two mediating effects, including the effect of remote mediation: NE → AWE → SA → TERB (β = 0.023, *p*-value < 0.01). In addition, awe partially mediates the effect of the perception of the natural environment on satisfaction (β = 0.072, *p*-value < 0.01), the perception of the availability of infrastructure on satisfaction (β = 0.038, *p*-value < 0.01), and the perception of the availability of infrastructure on TERB (β = 0.035, *p*-value < 0.05), respectively. The results confirmed the mediation effect of awe between stimuli and response factors in the proposed conceptual framework.

**TABLE 5:** Hypotheses tests of the mediating effects.

Hypothesis	Path	Original sample	Standard error	*t*- value	*p*- value	Support
H9	NE → AWE → SA	0.072	0.023	3.088	0.002	Yes
	AI → AWE → SA	0.038	0.015	2.634	0.008	Yes
H10	NE → AWE → TERB	0.065	0.023	2.791	0.005	Yes
	AI → AWE → TERB	0.035	0.015	2.306	0.021	Yes
NE → AWE → SA → TERB		0.023	0.008	2.860	0.004	Yes

*NE, the perception of the natural environment; AI, the perception of the availability of infrastructure; AWE, the experience of awe; SA, tourists’ satisfaction; TERB, tourists’ environmentally responsible behavior.*

## Discussion

The present study aimed to deepen our understanding of the relationships between the perception of the external factors of destinations (natural environment and availability of infrastructure), the emotion of awe, tourists’ satisfaction, and TERB. First, the findings of this study demonstrate that the perception of the natural environment positively influenced the awe and satisfaction of tourists (H1 and H2 were supported), but the positive effect of the natural environment on TERB was not found to be statistically significant (H3 was not supported). The tourists’ perceptions of the natural environment were shown to have a positive effect on their levels of awe and satisfaction, which is consistent with logic and with the findings reported in the literature (Lu et al., 2017; Pearce et al., 2017). Therefore, in our study, the extraordinary scenery of the destination directly affected the tourists’ satisfaction. The tourists’ perception of the vastness of the natural environment was shown to be an important precondition for eliciting awe.

Notably, in the present study, the natural environment was found to have no direct positive effect on TERB, thereby contradicting the findings of a previous study (Stewart and Craig, 2001). This discrepancy may be due to the differences in the degree of connection between individuals and the natural environment in different studies. The study by Stewart and Craig (2001) showed that individuals who are often exposed to the natural environment are more likely to adopt environmental protection behaviors. However, in the present study, the tourists’ perception of the natural environment was based on infrequent and occasional contact with nature at the destination. This connection is not enough to prompt tourists to implement ERB directly. For this to happen, an emotional connection must be established between the tourists and the natural environment (Vaske and Kobrin, 2001). Therefore, in this study, although the natural environment did not directly affect the behavior of the tourists, the perception of the natural environment elicited their awe and stimulated TERB through the mediating role of the awe connection.

Second, the perception of the availability of infrastructure was found to be an important antecedent variable of awe, satisfaction, and TERB (H4, H5, and H6 were supported). The positive relationship between the perception of the availability of infrastructure and awe is consistent with and constitutes an extension of the findings of Su and Swanson (2017) and Su L. et al. (2020) that the ecological and environmental practices adopted in particular destinations had an impact on tourists’ positive emotions. Our study confirmed that when tourists perceived the availability of pro-environment information, programs, products, and the facilities of the destination, the response was a specific positive emotion, a sense of awe. We suggest that the concern and emphasis on the environment underlying these environmental management practices were perceived by the tourists, and the destination’s eco-friendly reputation was recognized by the tourists, in turn, triggering their feelings of awe.

Moreover, the findings also suggest that the availability of infrastructure and satisfaction were positively correlated, which is consistent with the findings of Walsh et al. (2011) and Su L. et al. (2020). It is known that tourists’ satisfaction with their travel experience is affected by the main attributes of the destination, including the availability of destination facilities. Some environmental protection facilities, such as (recycle) bins and public transport, belong to the necessary infrastructure of the destination. If the destination cannot provide these infrastructural features, it is difficult for tourists to have a satisfactory experience. In other words, more available facilities, including environmental facilities, help to enhance tourists’ satisfaction. Also, the availability of suitable infrastructure was shown to have a positive impact on TERB, which is consistent with the findings of Chubchuwong et al. (2015) and Miller et al. (2015), whereby, if tourists consider that structural constraints preventing environmentally friendly facilities no longer exist, and that suitable facilities and services are provided, the objective conditions for implementing TERB are guaranteed. As a result, they are more likely to act responsibly toward the environment.

Third, according to the findings of our study, awe significantly influenced the tourists’ satisfaction and ERB (H7 and H8 were supported), in line with the findings of previous studies (Lu et al., 2017; Wang and Lyu, 2019). When tourists experience awe in a destination, they will be more satisfied with their tourism experience, and they will act more responsibly toward the environment. These findings extend the important role of positive emotion on tourist satisfaction (Su and Hsu, 2013) and TERB (Su L. et al., 2020), thereby supporting the importance of awe in tourists’ experiences and in destination development (Coghlan et al., 2012).

Finally, the results also confirm that awe is an emotional organism (O) that plays an important mediating role between stimuli and responses (H9 and H10 were supported). In light of the above, the direct relationship between stimuli and responses is not always consistent, but the awe experienced by tourists has always played a stable mediating role in that relationship. As proposed by the SOR framework, the stimuli can only change the responses of tourists after psychological changes (Su L. et al., 2020), such as the tourists’ experience of awe, have been induced. In the present study, the basic external factors of the destination (natural environment and availability of infrastructure) induced the tourists’ emotional responses (awe) and resulted in satisfaction with the overall tourist experience (satisfaction) and positive behaviors (TERB). The above results of this mediating effect reaffirm that it is essential to increase the levels of awe experienced by tourists.

## Conclusion

This study attempted to explore how the emotion of awe explains the influence of external factors of a destination on tourists’ levels of satisfaction and TERB. The empirical results showed that the direct relationships, with the exception of the link between the perception of the natural environment and TERB, were all supported. The mediating effects of awe were confirmed. This indicates that the model based on awe as a psychological organism and on the SOR framework can effectively explain the formation mechanism of satisfaction and TERB. These findings are important for academic research in the field of tourism and environmental psychology and also suggest useful implications for the management and development of particular destinations.

### Theoretical Significance

Based on the above results, this study contributes to the existing TERB literature through an integrated model built on the SOR framework as an innovative extension. The theories applied in existing studies to explain the formation mechanism of TERB have concentrated on tourists’ personal psychological and social factors. However, the contextual factors of the destination are often neglected. In the present study, building on the SOR theory, we have filled this gap in our attempt to understand the formation mechanism of TERB and the contextual factors affecting it. We anticipate that this extension of the application of the SOR framework will enrich future theoretical literature relating to TERB.

The introduction of the concept of awe and the verification of awe as an organism playing a mediating role in the relationship between stimuli and responses mean that this study provides not only a new theoretical perspective for research into the antecedent variables of TERB but also an effective supplement to awe research in the field of tourism. As a type of positive emotion, awe is often overlooked and has received less attention than other research topics in tourism literature (Pearce et al., 2017). Few tourism studies have specifically examined the relationship between awe and tourists’ attitudes and their behavioral intentions (Lu et al., 2017; Wang and Lyu, 2019; Su X. et al., 2020), especially on the effect of tourists’ perception of awe on TERB through on-site surveys. Such a study would extend the existing literature of tourists’ perception of awe and would contribute to the wider application of the findings of research into awe.

The effect of the availability of infrastructure on tourists’ experiences of awe was empirically tested for the first time in the present study, thereby enriching the theoretical research on the factors affecting tourists’ experiences of awe. Previous studies concentrated on how nature, religious and spiritual beliefs (Lu et al., 2017), artistic creation (Keltner and Haidt, 2003), and artificial landscapes (Wang and Lyu, 2019) elicit the experience of awe in individuals and identified three types of “awe elicitors” (tangible, sociocultural, and cognitive) (Keltner and Haidt, 2003). The perceived availability of infrastructure, as evidence of recognition and evaluation of the environmental management and the services provided by a destination, is not only a tangible factor enabling tourists to feel nature-induced awe but also a cognitive factor emphasizing the vastness and importance of nature. The result can be a feeling of the diminishment of an individual. It calls on tourists’ capacity for cognitive accommodation and directly inspires a sense of awe (Keltner and Haidt, 2003; Su and Swanson, 2017). However, to the best of our knowledge, no studies have examined the impact of the availability of infrastructure as a stimulus for experiencing awe. Therefore, our proposition, namely, that the availability of infrastructure acts as a stimulus and that awe acts as an organism, will enrich theoretical research on the characteristics of awe and the factors that affect it.

### Practical Implications

With regard to the practical implications, the findings of our study provide the most direct and effective way forward for the sustainable development of tourist destinations, a way to improve satisfaction and stimulate the implementation of tourists’ ERB by focusing on the controllable stimulus factors of a destination.

First, according to the coefficients of the influence path, the perception of the natural environment has a higher effect on tourists’ awe and satisfaction, indicating that it is a key factor in eliciting a sense of awe and meeting tourists’ needs. Therefore, enhancing tourists’ evaluation of the natural environment is essential and crucial for successful tourist destination management. The tourism administration department of nature-based destinations should provide tourists with easy access to nearby vantage points where they can experience the vast natural scenery. For example, reasonably located viewing platforms enable visitors to have a good view of the mountain scenery. Updated weather forecasts can ensure that tourists do not miss the sunrise, icy landscapes, snowy scenes, and other natural wonders, often the primary motivation for a visit. Tourists’ perception of beautiful natural scenery can also be enhanced and supplemented from multiple perspectives through guidebooks, videos, and even augmented reality experiences in the scenic area, thereby inducing awe and effectively increasing tourists’ satisfaction.

Second, the findings demonstrate that the perception of the availability of infrastructure at the destination is the key direct driver of TERB. An effective and feasible way for managers to promote tourists’ ERB is to remove the barriers to facilities. On the one hand, it is necessary to provide tourists with sufficient and available fundamental environmental protection facilities, such as environment-friendly transport, products, bins, and toilets. These facilities can not only facilitate tourists to visit to help them to experience awe but, more importantly, they can reduce the frequency of tourists’ unfriendly environmental behaviors, thereby indirectly or directly enhancing tourists’ TERB. On the other hand, information and services should be provided to advise tourists on “why to protect,” “how to protect,” and “what to do to protect,” thereby stimulating their actual participation in the environmental protection work. Tourists and their families who actively participate in scenic resources and environmental protection can enjoy preferential policies such as reduced ticket prices or exemptions designed to encourage their positive behaviors.

Third, our results also show that tourists’ emotional experiences, especially of awe, have an important effect on satisfaction and TERB, which should be of particular interest to tourism managers. To increase the awareness of awe by tourists, two basic characteristics of prototype theory that relate to the factors that inspire awe, namely, vastness and accommodation (Keltner and Haidt, 2003), must be taken into account. These factors should be incorporated into the design and development of the elements that create an attractive destination. In addition, destination management organizations should consider other socially contextual factors of the destination (Wang and Lyu, 2019), such as the perception of the availability of infrastructure as proposed in this study. This perception makes tourists’ visits feasible, and therefore, tourists can be exposed to a dramatic landscape and can experience awe. Notably, in a nature-based destination that also incorporates cultural resources, the studied area could promote the religious atmosphere (Lu et al., 2017), the perceived authenticity (Wang E. et al., 2020), the involvement (Su X. et al., 2020), and other variables based on the resource characteristics to stimulate tourists’ sense of awe and ultimately, to stimulate their responses such as satisfaction, loyalty, and ERB.

Furthermore, destination management should consider the carrying capacity of the popular scenic spots in the area and avoid over-development problems caused by convenient facilities (e.g., a convenient and environmentally friendly transport system). This action would limit the degree of damage and maintain the quality of the tourism experience. For example, the ecological environment carrying capacity analysis and warning system can be activated to conduct real-time analysis of a real-time number of visitors, visitor routes, preferred scenic spots, and other aspects. Based on the results of the analysis, the managers can promptly adopt measures such as tourist flow restriction and diversion, opening up new tourist routes, and adjusting opening times to improve the visitors’ evaluation of their experience. It is noteworthy that in the marketing of natural scenic spots similar to the case in this study, managers should avoid the impact of high expectation promotional materials on tourist satisfaction. For example, sometimes, due to bad weather, some tourists cannot reach the peak because the environmentally friendly vehicles are not running. Zhurong Peak, which has ice and snow, is the main marketing attraction in winter. Tourists who are not able to walk to the peak may not have a satisfactory travel experience. Taking this possibility into consideration, marketing materials should warn that this might be a possible outcome of a visit to Zhurong Peak. Alternatively, managers must carefully consider feasible solutions to potential obstacles, thereby helping tourists to be in close proximity to natural wonders.

Finally, based on the findings of this study, faced with environmental pressures and the difficulties of maintaining the ecological environments of destinations, managers need to cultivate positive emotional responses by optimizing the design and construction of destination environments (Mair and Laing, 2013) and, ultimately, by delivering memorable and satisfying experiences to visitors and promoting their pro-environmental behaviors. It is true that to better guide and facilitate tourist behavior, destination planners and developers should also consider other feasible and straightforward methods. For example, while providing environmental protection facilities, the destination should also explain and demonstrate the importance of environmental protection and the negative consequences of destroying the environment, respectively, that is, through environmental education and explanation (Gao et al., 2018), help inspire tourists’ social awareness of responsibility (Luo et al., 2020). In addition, managers can also emphasize reward and punishment strategies for different behaviors in the scenic area (Li and Chen, 2019), thereby correspondingly triggering positive behaviors of tourists and reducing their deviant behaviors. All these direct and indirect management practices will contribute to the environmental sustainability of TERB and the destination.

## Limitations and Future Research

Several limitations of this study should be noted and resolved in future studies. The first limitation is the lack of generalizability of the research results. The present study chose a mountain-based natural destination in mainland China as the location for collecting data to test the relationships between the variables. The different cultural backgrounds and the attractions of the destination might make replication of this research, built on the SOR framework, in the context of other destinations challenging. Researchers would still need to consider the environmental stimulus characteristics of destinations and the perception of tourists’ experiences to confirm the validity of the findings. Furthermore, as the study design and data collection were pre-pandemic, the impact of COVID-19 was not considered in this study. Its impact need be further studied in the future. As the COVID-19 pandemic persists, there may be greater awareness of the importance of environmental responsibility, which can influence tourists’ behavior. Whether the pandemic will affect tourists’ attitude toward environmental responsibility deserves further study.

Another limitation of this study is that the experience of awe was examined only in the context of a nature-based mountain tourism destination. In the future, it will be necessary to study tourists’ experience of awe and the factors that affect it in other settings, such as historical destinations, high-tech tourist destinations, and the residences of celebrities. Since tourists’ experience of awe can encourage them to prolong, remember, or relive past experiences, resulting in a sense of a place (Coghlan et al., 2012), future studies can further explore the relationships between place attachment and tourists’ memorable experiences of awe, with a view to their eudemonic value and contribution to well-being of individuals in their daily lives.

Some concepts, such as perceived value, are advocated to contain affective, social, hedonic, and utilitarian dimensions. Those are essential for building positive emotions that lead to tourist satisfaction. Perceived value is considered a reliable concept to better explain behavior (Kim and Thapa, 2018). Consequently, some consideration should be given in follow-up research to building a comprehensive model incorporating perceived value, specific positive emotions, and tourist attitudes to better understand tourist behavior. TERB can also extend to their everyday life situations (Ramkissoon et al., 2012). The broader social environmental value this could create deserves special exploration in the future.

Finally, the emotion of awe elicited by the natural landscape in our study is a component of the sublime emotion in response to nature (Bethelmy and Corraliza, 2019). We recommend further research on the role of the sublime as an antecedent to the tourism experience and to potential tourist behaviors, and its relation to awe in various tourism contexts (e.g., dark tourism).

## Data Availability Statement

The original contributions presented in the study are included in the article/Supplementary Material, further inquiries can be directed to the corresponding author/s.

## Ethics Statement

Ethical review and approval was not required for the study on human participants in accordance with the local legislation and institutional requirements. Written informed consent for participation was not required for this study in accordance with the national legislation and the institutional requirements.

## Author Contributions

JJ and BG conceived the study. JJ and XS collected and analyzed the data. JJ, BG, and XS wrote the manuscript. All authors designed the study, read and approved the manuscript, and agreed to be accountable for all aspects of the work.

## Conflict of Interest

The authors declare that the research was conducted in the absence of any commercial or financial relationships that could be construed as a potential conflict of interest.

## Publisher’s Note

All claims expressed in this article are solely those of the authors and do not necessarily represent those of their affiliated organizations, or those of the publisher, the editors and the reviewers. Any product that may be evaluated in this article, or claim that may be made by its manufacturer, is not guaranteed or endorsed by the publisher.

## Footnotes

1
https://scholar.google.com/
